# Maternal immune activation by poly(I:C) induces expression of cytokines IL-1β and IL-13, chemokine MCP-1 and colony stimulating factor VEGF in fetal mouse brain

**DOI:** 10.1186/1742-2094-9-83

**Published:** 2012-04-30

**Authors:** Juan L Brusés

**Affiliations:** 1 Department of Anatomy and Cell Biology, The University of Kansas School of Medicine, Kansas City, KS, 66160, USA; 2 Department of Psychiatry and Behavioral Sciences, The University of Kansas School of Medicine, Kansas City, KS, 66160, USA; 3 Department of Anatomy and Cell Biology, University of Kansas School of Medicine, 3901 Rainbow Boulevard MS 3038, Kansas City, KS, 66160, USA; 4 Present address: Universite Joseph Fourier, Institut Jean Roget, LAPM - UMR5163 - CNRS, Domaine de la Merci, 38700, La Tronche, France

**Keywords:** CNS development, Immune response-associated secreted factors, Innate immune response, Maternal viral infection, Psychiatric disorders

## Abstract

**Background:**

Maternal viral infection during pregnancy is associated with an increase in the incidence of psychiatric disorders with presumed neurodevelopmental origin, including autism spectrum disorders and schizophrenia. The enhanced risk for developing mental illness appears to be caused by deleterious effects of innate immune response-associated factors on the development of the central nervous system, which predispose the offspring to pathological behaviors in adolescence and adulthood. To identify the immune response-associated soluble factors that may affect central nervous system development, we examined the effect of innate immune response activation by polyriboinosinic-polyribocytidylic acid (poly(I:C)), a synthetic analogue of viral double-stranded RNA, on the expression levels of pro- and anti-inflammatory cytokines, chemokines and colony stimulating factors in fetal and postnatal mouse brain 6 h and 24 h after treatment.

**Methods:**

C57BL/6J pregnant mice (gestational day 16) or newborn mice (postnatal day 4) received a single intraperitoneal injection of the synthetic analogue of viral double-stranded RNA poly(I:C) (20 mg/kg). Thirty-two immune response-associated soluble factors, including pro- and anti-inflammatory cytokines, chemokines and colony stimulating factors, were assayed 6 h and 24 h after poly(I:C) injection using multiplexed bead-based immunoassay (Milliplex Map) and processed in a Luminex 100 IS instrument.

**Results:**

Maternal exposure to poly(I:C) at gestational day 16 induced a significant increase in cytokines interleukin (IL)-1β, IL-7 and IL-13; chemokines monocyte chemoattractant protein 1 (MCP-1), macrophage inflammatory protein (MIP)-1α, interferon gamma-induced protein (IP)-10 and monokine induced by IFN-gamma (MIG); and in the colony stimulating factor vascular endothelial growth factor (VEGF) in the fetal brain. IL-1β showed the highest concentration levels in fetal brains and was the only cytokine significantly up-regulated 24 h after maternal poly(I:C) injection, suggesting that IL-1β may have a deleterious impact on central nervous system development. In contrast, poly(I:C) treatment of postnatal day 4 pups induced a pronounced rise in chemokines and colony stimulating factors in their brains instead of the pro-inflammatory cytokine IL-1β.

**Conclusions:**

This study identified a significant increase in the concentration levels of the cytokines IL-1β and IL-13, the chemokine MCP-1 and the colony stimulating factor VEGF in the developing central nervous system during activation of an innate immune response, suggesting that these factors are mediators of the noxious effects of maternal immune activation on central nervous system development, with potential long-lasting effects on animal behavior.

## Background

The etiology of psychiatric disorders with presumed neurodevelopmental origin, including autism spectrum disorders and schizophrenia, remains largely unknown. However, epidemiological and genetic studies suggest that the interaction between genetic abnormalities and epigenetic factors perturb the formation of the central nervous system (CNS), resulting in the cognitive, sensorial and emotional dysfunctions observed in mental illness [1-5]. One important epigenetic risk factor for autism and schizophrenia is the occurrence of maternal infection during pregnancy [2,6-12], suggesting that the increased risk for mental illness arises from the effect of the innate immune response (for example, cytokines) on CNS formation [2,13,14].

Immune response-associated secreted factors (IRSF) released during the activation of an innate immune response (cytokines, chemokines and colony stimulating factors (CSF)) modulate various aspects of neural development, including neuronal survival, differentiation and growth [15-20]. This evidence suggests that abnormal activation of IRSF-mediated signaling affects CNS development and increases the risk for developing psychopathology. However, evidence for a direct association between the effect of a particular IRSF on brain development and the appearance of behavioral abnormalities has only recently started to be established [21,22].

The synthetic analogue of viral double-stranded RNA (dsRNA) polyriboinosinic-polyribocytidylic acid (poly(I:C)) mimics the host acute innate immune response to viral infection [23-25]. Treatment of pregnant mice with poly(I:C) alters cytokine expression levels in the fetal brain and causes behavioral abnormalities in post-pubertal offspring [21,22,26-29]. These studies have established an experimental mouse model of mental disorders in which psychopathological conditions are triggered by maternal innate immune activation during pregnancy [2,30-32]. However, to elucidate the mechanisms responsible for the deleterious effects of IRSF on brain development, it is necessary to identify the cytokines, chemokines and CSF that are regulated in the developing brain by innate immune activation. To this end, we carried out a comprehensive analysis of 32 IRSF in the fetal brain and maternal serum of mice after maternal innate immune activation by poly(I:C). In addition, to determine whether the maternal environment affects the expression of IRSF in the developing brain during pregnancy, we examined the effects of innate immune response activation in early postnatal life on IRSF expression levels in the brains of newborn pups treated with poly(I:C). This analysis revealed that maternal immune activation induces the expression of a specific subset of cytokines in the developing fetal brain that may contribute to long-lasting functional abnormalities.

## Methods

### Animals

Female and male C57BL/6J mice breeders (10 to 12 weeks old) were obtained from Jackson Laboratories (The Jackson Laboratory, Bar Harbor, ME, USA) and kept in a pathogen-free facility. After two weeks of acclimatization, animals were bred to establish an in-house pathogen-free colony. Crosses were set in the evening and the next morning successful copulation was verified by the presence of a vaginal plug; this was considered gestational day (GD) 0. Animals were maintained in a 14 h light and 10 h dark cycle with the lights turning on at 6 a.m. and off at 8 p.m. Handling and manipulation of the animals used in this study has been approved by the Institutional Animal Care and Use Committee of the University of Kansas School of Medicine.

### Drug treatments

Pregnant dams on GD16 were weighed and received a single intraperitoneal (i.p.) injection of poly(I:C) sodium salt, (20 mg/kg; Sigma-Aldrich, St. Louis, MO, USA) reconstituted in approximately 100 μL of sterile and endotoxin-free phosphate-buffered saline (PBS), pH 7.2 (Sigma-Aldrich) in pyrogen-free tubes, injected using a 27 G1/2 needle under aseptic conditions. Pregnant animals injected with 100 μL of PBS only were used as control. Four-day-old pups from untreated mothers were weighed and received an i.p. injection of poly(I:C) (20 mg/kg) or PBS. Animals were injected at 10 a.m. A dose of 20 mg/kg on GD16 was chosen because previous studies have shown that this treatment caused long-lasting effects on behavioral performance and the appearance of behaviors associated with psychiatric symptoms [21,33]. Postnatal day (PND) 4 was chosen because at this developmental stage formation of neural circuits is not completed [34-36] and, therefore, the CNS is vulnerable to epigenetic insults, including viral infections.

### Tissue harvest and processing

Mice were deeply anesthetized 6 or 24 h after injection by exposure to isoflurane gases (IsoFlo, Abbott, Abbott Park, IL, USA) and killed by cervical dislocation. Maternal blood was collected via cardiac puncture, transferred to 1.5 mL centrifuge tubes and allowed to clot at room temperature for 1 h. Fetuses were surgically delivered and carefully examined for injuries that might have been caused during the injection and manipulation of the animals. An average of 10 pups per pregnant dam was obtained. Pregnant animals with less than six pups were not used for the study. Fetuses were killed by decapitation and heads placed in ice-cold PBS for immediate harvesting of the brains. Using a stereomicroscope, the brain was quickly removed, the meninges and subarachnoid vasculature were pilled off, and the brain weighed and homogenized in 5 vol (w/v; approximately 400 μL) of ice-cold homogenization buffer (0.05 % Tween 20, 10 mM sodium fluoride and complete ethylenediaminetetraacetic acid-free protease inhibitor cocktail (Roche, Basel, Switzerland)), in Dulbecco’s PBS, pH 7.2 (GIBCO, Invitrogen, Carlsbad, CA, USA). Tissue was homogenized in a Teflon/glass homogenizer on ice followed by 10 sec of ultrasonication at 2 W. Blood and brain homogenates were centrifuged at 20,000 × g for 10 min at 4°C, the supernatants transferred to a new tube, aliquoted, and stored at −80°C. The tail from each embryo was removed and stored at −80°C for DNA extraction and sex identification. Total protein concentration in serum and brain homogenates was determined by bicinchoninic acid protein assay (Pierce, Rockford, IL, USA). A concentration of 6 to 10 μg/μL of total protein was measured in each sample. PND5 pups were deeply anesthetized by exposure to isoflurane gases, placed on ice and killed by decapitation. The brains were removed and processed as described above.

### Immune response-associated secreted factors measurements and data analysis

Sera and tissue homogenates were assayed for cytokines, chemokines and CSF using the multiplexed bead-based immunoassay Milliplex Map, MPXMCYTO70KPMX32 (Millipore, Billerica, MA, USA), which simultaneously detects mouse cytokines interleukin (IL)-1α, IL-1β, IL-2, IL-3, IL-4, IL-5, IL-6, IL-7, IL-9, IL-10, IL-12 (p40), IL-12 (p70), IL-13, IL-15, IL-17, intereferom (IFN)-γ, tumor necrosis factor (TNF)-α and leukemia inhibitory factor (LIF); chemokines eotaxin, monocyte chemotactic protein-1 (MCP-1), macrophage inflammatory protein (MIP)-1α, MIP-1β, regulated on activation normal T cells expressed and secreted (RANTES), interferon inducible protein 10 (IP-10), keratinocyte derived chemokine (KC), lipopolysaccharide (LPS) induced CXC chemokine (LIX), monokine induced by gamma-interferon (MIG) and MIP-2; and CSF granulocyte-macrophage (GM)-CSF, granulocyte (G)-CSF, macrophage (M)-CSF and vascular endothelial growth factor (VEGF). These three families of secreted proteins are here referred as IRSF.

The Milliplex Map assay is a Luminex bead-based immunoassay that uses premixed beads coated with antibodies that recognize a panel of 32 analytes in each sample. IRSF assays were performed in 96-well plates according to the manufacturer’s instructions. Briefly, each assay plate layout consisted of six standards in duplicate; two positive controls in duplicate, two blank wells and up to 78 tissue samples. At the time of the assay, samples were thawed on ice and clarified by centrifugation at 20,000 × g for 10 min at 4°C and the supernatant used for the analysis. Serum samples were diluted in serum sample diluent provided by the immunoassay kit and brain homogenates samples were diluted in homogenization buffer. Each well was loaded with 300 μg of total proteins from serum samples or 150 μg of total protein from brain homogenates samples. Each tissue sample was run in triplicate in the same plate. Samples from poly(I:C)- and PBS-treated animals were analyzed in the same plate. Samples and standards were processed using the Luminex 100 IS instrument platform and related Luminex 100 IS software (version 2.3; Luminex Corporation, Austin, TX, USA). The readouts were analyzed with the standard version of the Millplex Analyst software (Millipore). A five-parameter logistic regression model with weighting was used to create standards curves (pg/mL) and calculate the mean of sample concentration from each triplicate.

The maximum level of detection for each factor ranged from 7,000 to 10,000 pg/mL in all samples. The minimum level of detection (MinDC) was slightly different between samples depending on whether the sample was from serum or brain homogenates. MinDC values in brain homogenates were: cytokines, IL-1α 2.6 pg/mL, IL-1β 0.3 pg/mL, IL-2 0.4 pg/mL, IL-3 1.2 pg/mL, IL-4 0.3 pg/mL, IL-5 1.0 pg/mL, IL-6 2.6 pg/mL, IL-7 1.3 pg/mL, IL-9 8.6 pg/mL, IL-10 1.2 pg/mL, IL-12(p40) 2.8 pg/mL, IL-12(p70) 1.7 pg/mL, IL-13 3.7 pg/mL, IL-15 3.7 pg/mL, IL-17 0.4 pg/mL, IFN-γ 1.6 pg/mL, TNF-α 1.7 pg/mL and LIF 0.8 pg/mL; CSF, GM-CSF 0.2 pg/mL, G-CSF 1.8 pg/mL, M-CSF 1.5 pg/mL and VEGF 0.1 pg/mL; chemokines, eotaxin 1.6 pg/mL, MCP-1 0.1 pg/mL, MIP-1α 2.2 pg/mL, MIP-1β 3.9 pg/mL , RANTES 1.7 pg/mL, IP-10 1.0 pg/mL, KC 1.4 pg/mL, LIX 0.4 pg/mL, MIG 2.3 pg/mL and MIP-2 1.9 pg/mL. MinDC values in serum samples were: cytokines, IL-1α 0.5 pg/mL, IL-1β 0.6 pg/mL, IL-2 0.4 pg/mL, IL-3 1.3 pg/mL, IL-4 0.4 pg/mL, IL-5 0.3 pg/mL, IL-6 0.4 pg/mL, IL-7 0.4 pg/mL, IL-9 0.3 pg/mL, IL-10 1.9 pg/mL, IL-12(p40) 1.2 pg/mL, IL-12(p70) 0.3 pg/mL, IL-13 0.4 pg/mL, IL-15 0.4 pg/mL, IL-17 0.3 pg/mL, IFN-γ 1.6 pg/mL, TNF-α 1.2 pg/mL and LIF 1.2 pg/mL; CSF, GM-CSF 0.4 pg/mL, G-CSF 0.4 pg/mL, M-CSF 0.4 pg/mL and VEGF 0.9 pg/mL; chemokines, eotaxin 0.3 pg/mL, MCP-1 0.4 pg/mL, MIP-1α 0.2 pg/mL, MIP-1β 4.5 pg/mL, RANTES 1.1 pg/mL, IP-10 0.8 pg/mL, KC 1.2 pg/mL, LIX 3.4 pg/mL, MIG 0.1 pg/mL and MIP-2 1.0 pg/mL. Positive control values were reproducible between assays and always fell within the accepted recovery of 80 to 120 % of expected values. Samples exhibiting a coefficient of variation >15 % were omitted from final data analysis. Tissue and serum sample values obtained in pg/mL were normalized to pg/100 μg of total protein throughout the study to facilitate comparisons between groups.

### Sex determination

Genomic DNA isolated from the tail was used to determine the gender of each pup by PCR using primers annealing to the X chromosome *DXMit26* gene (X-forward, 5′TTGGCAAGCATG CTTTACTG3′; X-reverse, 5′AGG AACATGGAAACACCTGC3′), resulting in a product of 220 bp, and to the Y chromosome gene *zinc finger Y-chromosomal protein-1* gene (Y-forward, 5′CTCCTGATGGACAAACTTTAC3′; Y-reverse, 5′TGAGTGCTGATGGGTGACGG3′) resulting in a product of 400 bp. No statistically significant differences were observed between genders from the same treatment group; therefore, a similar number of samples from each sex was pooled together for the study.

### Statistical analysis

Statistical comparisons between treatment groups were carried out with non-directional Student’s *t* tests using Excel XP. Statistical significance was set at *P* <0.05.

## Results

### Poly(I:C) induces a broad increase in immune response-associated factors in maternal serum

To examine the effects of maternal innate immune activation during pregnancy on IRSF expression levels, a multiplexed bead-based assay (Milliplex Map Assay, Millipore) was performed and a Luminex 100 instrument was used for analysis. The multiplex bead-based assay simultaneously detects 32 IRSF covering a wide spectrum of pro- and anti-inflammatory cytokines (IL-1α, IL-1β, IL-2, IL-3, IL-4, IL-5, IL-6, IL-7, IL-9, IL-10, IL-12 (p40), IL-12 (p70), IL-13, IL-15, IL-17, IFN-γ, TNF-α and LIF), chemokines (eotaxin, MCP-1, MIP-1α, MIP-1β, RANTES, IP-10, KC, LIX, MIG and MIP-2) and CSF (GM-CSF, G-CSF, M-CSF and VEGF), with a low-end sensitivity of detection ranging from 0.2 to 4 pg/mL (see Methods). These three classes of IRSF are involved in the first steps of the antiviral innate immune response and promote the development and trafficking of various subsets of immune and non-immune cells. Thus, this analysis provides a comprehensive overview of the host reaction to the foreign agent.

To induce maternal innate immune activation, pregnant mice (C57BL/6J, 12 to 14 weeks old) received a single i.p. injection of the synthetic analogue of viral dsRNA poly(I:C) (20 mg/kg) in 100 μL of PBS [37] on GD16. This dose of poly(I:C) causes long-lasting behavioral abnormalities in the progeny [33]. DsRNA represents a molecular pattern associated with diverse types of viral infections because it is produced by most viruses during their replication cycle within the host. Poly(I:C) is recognized primarily by Toll-like receptor 3 (TLR3), a member of the family of innate immune-recognition receptors that recognize molecular patterns associated with viral pathogens and induce an antiviral innate immune response [23,24,38]. Animals were killed 6 h or 24 h after injection and maternal blood and fetal brains were collected and processed for analysis with a multiplexed bead-based assay (see Methods). These two time-points were aimed at capturing early and late changes in IRSF expression levels. Age-matched pregnant mice injected with 100 μL of PBS were used as controls.

All IRSF assayed were detected in control maternal serum and a wide range of factors were up-regulated by poly(I:C) treatment 6 h after injection (Table 1). The most pronounced increases in cytokine expression levels were observed in IL-6 (5935 %), IL-12(p40) (789 %), IL-12(p70) (289 %), IL-13 (784 %), IL-15 (570 %), INF-γ (253 %), TNF-α (626 %) and IL-10 (1,210 %), many of which participate in the activation of the antiviral innate immune response [39]. In addition to the increase in cytokines, most chemokines and CSF analyzed were highly up-regulated (by 108 % to 23,700 %) 6 h after poly(I:C) treatment as compared to PBS-injected animals. By 24 h post-injection, most cytokine, chemokine and CSF expression levels had returned to control values or remained similar to the levels detected at 6 h after injection. This analysis indicates that i.p. administration of poly(I:C) triggered a broad antiviral maternal innate immune activation, resulting in a substantial increase in the concentration levels of the three types of IRSF involved in the innate immune response.

**Table 1:** Cytokine, chemokine and colony stimulating factor concentrations in prenatal maternal serum

	**6 h after injection**				**24 h after injection**			
	**Control**	**poly(I:C)**	**%** change	**↑ or ↓**	**Control**	**poly(I:C)**	**% change**	**↑ or ↓**
**Pro- and anti-inflammatory cytokines**								
IL-1α	386.3 ± 123.3	260.0 ± 1.9	38	↓	308.8 ± 90.0	160 ± 78.3	38	↓
IL-1β	3.6 ± 1.0	7.0 ± 0.2	97	↑*	1.5 ± 0.2	7.9 ± 5.1	736	↑
IL-2	0.7 ± 0.2	1.1 ± 0.2	70	↑	ND	0.4 ± 0.3	ND	↑
IL-3	0.5 ± 0.1	0.6 ± 0.3	26	↑	ND	0.8 ± 0.8	ND	↑
IL-4	0.1 ± 0.0	0.2 ± 0.3	25	↑	ND	0.6 ± 0.6	ND	↑
IL-5	8.5 ± 4.5	14.4 ± 1.4	69	↑	1.9 ± 0.6	3.8 ± 0.7	84	↑
IL-6	1.6 ± 0.1	96.6 ± 48.0	5935	↑	1.3 ± 0.3	5.4 ± 2.7	478	↑
IL-7	3.2 ± 1.3	1.4 ± 0.2	58	↓	3.5 ± 1.0	7.1 ± 6.1	305	↑
IL-9	20.0 ± 9.8	51.7 ± 3.8	158	↑*	12.6 ± 7.4	32.7 ± 24.6	216	↑
IL-12(p40)	5.3 ± 2.8	47.4 ± 10.3	789	↑*	2.4 ± 0.4	6.2 ± 3.4	214	↑
IL-12(p70)	3.2 ± 1.2	12.6	289	↑*	0.6 ± 0.3	8.4 ± 6.7	3236	↑
IL-13	30.7 ± 7.8	271.8 ± 19.9	784	↑**	44.5 ± 13.7	45.0 ± 14.9	3	
IL-15	52. ± 2.3	34.8 ± 5.1	570	↑**	3.4 ± 1.7	7.1 ± 4.0	116	↑
IL-17	2.0 ± 0.5	3.2 ± 0.8	63	↑	1.0 ± 0.1	2.9 ± 2.3	343	↑
IFN-γ	1.4 ± 04	4.8 ± 0.9	253	↑*	1.6 ± 0.1	4.3 ± 2.5	309	↑
TNF-α	1.9 ± 0.6	13.6 ± 1.5	626	↑**	1.2 ± 0.2	4.1 ± 1.7	266	↑
IL-10	4.0 ± 1.2	52.8 ± 7.2	1210	↑**	3.0 ± 0.2	10.4 ± 2.0	250	↑*
LIF	2.5 ± 1.1	4.3 ± 1.4	74	↑	ND	0.6 ± 0.0	ND	↑*
**Chemokines**								
Eotaxin	86.0 ± 26.6	333.3 ± 93.9	287	↑*	59.2 ± 10.1	70.0 ± 23.2	30	↑
MCP-1	5.4 ± 0.3	1154.0 ± 432.3	21400	↑*	5.5 ± 0.4	48.2 ± 24.9	976	↑
MIP-1α	7.4 ± 3.9	66.2 ± 5.5	802	↑**	1.8 ± 0.9	11.6 ± 5.2	559	↑*
MIP-1β	14.9 ± 5.0	456.0 ± 112.5	2986	↑*	5.1 ± 0.6	23.3 ± 7.3	533	↑
RANTES	6.9 ± 3.6	1640.7 ± 735.6	23700	↑*	5.0 ± 2.4	89.1 ± 27.3	569	↑*
IP-10	22.8 ± 3.7	2089.0 ± 518.5	9049	↑*	25.3 ± 4.6	180.6 ± 28.5	1476	↑*
KC	17.1 ± 2.9	64.3 ± 25.4	276	↑*	12.7 ± 2.8	15.1 ± 7.4	52	↑
LIX	1818.7 ± 598.1	1640.3 ± 481.8	10	↓	1451.0 ± 483.7	1277.7 ± 501.7	1	↑
MIG	34.7 ± 1.9	1855 ± 144.8	5259	↑**	75.8 ± 25.8	1932.1 ± 867.7	2012	↑*
MIP-2	46.5 ± 8.8	96.0 ± 1.7	108	↑**	25.3 ± 3.3	42.8 ± 17.5	168	↑
**Colony stimulating factors**								
GM-CSF	4.4 ± 0.3	12.1 ± 2.5	174	↑*	10.0 ± 6.1	6.9 ± 2.9	32	↓
G-CSF	165.7 ± 32.7	2906.7 ± 678.8	1654	↑*	109.7 ± 26.9	1823.6 ± 778.4	2003	↑*
M-CSF	2.0 ± 0.8	11.4 ± 1.9	468	↑*	0.8 ± 0.1	3.8 ± 1.9	465	↑
VEGF	0.5 ± 0.1	1.3 ± 0.0	185	↑**	0.5 ± 0.1	0.5 ± 0.1	6	↑

Finally, we examined whether circadian variations were observed in the maternal serum by comparing the concentrations of IRSF at 6 h with the ones obtained at 24 h after PBS injection. Treatments were carried out at 10 a.m.; therefore, the 6 h post-injection time-point occurred at 4 p.m. (close to the end of the light period), while the 24 h time-point occurred at 10 a.m. (close to the beginning of the light period). None of the IRSF detected at both time-points showed a significant difference between the two times of the day. All IRSF measured were detected in maternal serum at the 6 h time-point (4 p.m.). In contrast IL-2, IL-3 and IL-4 were undetectable at the 24 h time-point (10 a.m.) (Table 1); however, their concentrations at 4 p.m. were less than 1 pg/100 μg of protein, indicating that no significant variations occurred between the two time-points.

### Maternal innate immune activation up-regulates pro-inflammatory cytokines in fetal brain

We next examined the concentration levels of IRSF in the brain of the fetuses from pregnant females treated with PBS or poly(I:C) on GD16 and killed 6 h or 24 h after injection (Table 2). Care was taken to remove the meninges and superficial vasculature from the brains before homogenization to minimize the contribution of IRSF from blood and vessels. The concentration values were normalized to 100 μg of total protein from each sample. Various pro- and anti-inflammatory cytokines were detected in control brain samples including IL-1β, IL-7, IL-9, IL-13, IL-15, IL-17 and IL-10, indicating that cytokines are physiologically present in the developing brain without the induction of an innate immune response. The inflammatory cytokines IL-1β, IL-9 and IL-10 showed the highest levels of expression, ranging from 27 to 128 pg/100 μg of protein, while IL-7, IL-13, IL-15 and IL17 concentrations were below 10 pg/100 μg of protein. The chemokines eotaxin, MCP-1, MIP-1α, IP-10 and MIG were also present in brain homogenates from PBS-treated animals at concentrations ranging from 7 to 300 pg/100 μg of protein, while MIP-1β, RANTES and KC were below 4 pg/100 μg of protein. The CSF GM-CSF, M-CSF and VEGF were detected in control samples at very low concentrations (<4 pg/100 μg of total protein). In contrast to serum, in which all IRSF were detected, various cytokines (IL-1α, IL-2, IL-3, IL-4, IL-5, IL-6, IL-12(p40), IL-12(p70), IFN-γ, TNF-α and LIF), chemokines (LIX and MIP-2 ) and G-CSF were undetectable in brain homogenates despite the high sensitivity of the detection method, indicating that these factors are not present at detectable levels in the fetal brain under physiological conditions at this developmental stage. No differences in the concentration values were observed between genders in both the PBS- and poly(I:C)-treated groups (data not shown); therefore, a similar number of male and female mice were pooled together in each experimental group.

**Table 2:** Cytokine, chemokine and colony stimulating factor concentrations in prenatal brain homogenates

	**6h after injection**				**24h after injection**			
	**Control**	**poly(I:C)**	**% change**	**↑ or ↓**	**Control**	**poly(I:C)**	**% change**	**↑ or ↓**
**Pro- and anti-inflammatory cytokines**								
IL-1α	ND	ND	ND		ND	ND	ND	
IL-1β	43.9 ± 3.5	46.5 ± 9.6	6	↑	94.9 ± 5.3	126.9 ± 11.3	34	↑*
IL-2	ND	ND	ND		ND	ND	ND	
IL-3	ND	ND	ND		ND	ND	ND	
IL-4	ND	ND	ND		ND	ND	ND	
IL-5	ND	ND	ND		ND	ND	ND	
IL-6	ND	ND	ND		ND	ND	ND	
IL-7	1.2 ± 0.1	3.7 ± 0.9	205	↑*	3.4 ± 0.8	3.9 ± 1.7	23	↑
IL-9	101.9 ± 3.3	108.5 ± 5.6	7	↑	128.1 ± 3.1	125.3 ± 3.3	2	↓
IL-12(p40)	ND	ND	ND		ND	ND	ND	
IL-12(p70)	ND ±	ND ±	ND		ND	ND	ND	
IL-13	3.8 ± 0.9	17.3 ± 5.5	356	↑*	4.1 ± 0.7	9.6 ± 2.9	136	↑
IL-15	ND	ND	ND		9.7 ± 1.1	7.9 ± 1.0	19	↓
IL-17	ND	ND	ND		7.7 ± 0.5	7.1 ± 1.3	8	↓
IFN-γ	ND	ND	ND		ND	ND	ND	
TNF-α	ND	ND	ND		ND	ND	ND	
IL-10	27.7 ± 1.4	25.3 ± 2.5	9	↓	23.8 ± 1.6	27.5 ± 2.7	15	↑
LIF	ND	ND	ND		ND	ND	ND	
**Chemokines**								
Eotaxin	18.7 ± 1.1	22.5 ± 2.5	20	↑	35.0 ± 3.5	32.6 ± 2.8	6	↓
MCP-1	13.8 ± 1.5	25.0 ± 5.9	81	↑	6.8 ± 0.5	21.7 ± 6.9	218	↑*
MIP-1α	300.3 ± 8.7	261.5 ± 9.7	13	↓**	153.6 ± 11.7	280.0 ± 47.7	82	↑**
MIP-1β	ND	ND	ND		3.4 ± 0.5	2.3 ± 0.6	31	↓
RANTES	ND	ND	ND		1.5 ± 0.1	1.7 ± 0.5	14	↑
IP-10	23.2 ± 2.1	60.5 ± 3.4	160	↑**	26.3 ± 2.1	41.1 ± 3.5	56	↑**
KC	ND	ND	ND		1.8 ± 0.1	1.7 ± 0.1	4	↓
LIX	ND	ND	ND		ND	ND	ND	
MIG	11.3 ± 2.9	17.2 ± 2.2	52	↑*	49.0 ± 2.4	39.3 ± 3.3	20	↓*
MIP-2	ND	ND	ND		ND	ND	ND	
**Colony stimulating factors**								
GM-CSF	ND	ND	ND		2.4 ± 0.7	1.3 ± 0.6	47	↓
G-CSF	ND	ND	ND		ND	ND	ND	
M-CSF	ND	ND	ND		3.7 ± 0.1	2.9 ± 1.1	21	↓
VEGF	0.9 ± 0.0	2.4 ± 0.5	177	↑*	1.8 ± 0.2	1.9 ± 0.3	7	↑

**Figure 1:**
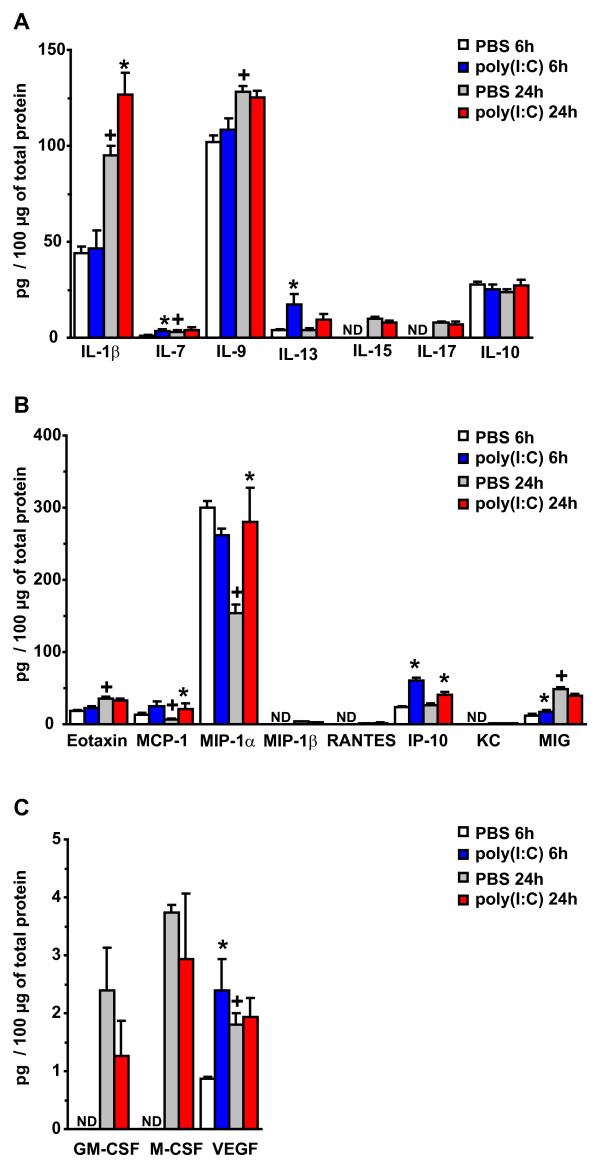
**Analysis of immune response-associated soluble factors expression in fetal brain homogenates after maternal innate immune activation.** Pregnant mice received a single i.p. injection of poly(I:C) or PBS on GD16 and were killed 6 h and 24 h after treatment. IRSF concentrations in fetal brain homogenates were determined by multiplexed bead-based immunoassay (Milliplex Map). Only factors detected at least at one time-point are plotted. (**A**) Comparison of the expression levels of pro- and anti-inflammatory cytokines between saline and poly(I:C) injected animals. IL-1α, IL-2, IL-3, IL-4, IL-5, IL-6, IL-12(p40), IL-12(p70), IFN-γ, TNF-α and LIF were not detected at both time-points. At 6 h after treatment, IL-7 and IL-13 showed significantly higher concentrations as compared to PBS-treated animals. At 24 h post-treatment, only IL-1β showed significantly higher expression levels compared to saline-treated animals. Circadian variations in control animals in the concentration levels of IL-1β and IL-9 were also detected. (**B**) Comparison of chemokine expression levels showed increased concentration levels of IP-10 and MIG 6 h after treatment while MCP-1, IP-10 and MIP-1α were increased at 24 h post-treatment. Significant changes in control animals between the two times of the day were observed for eotaxin, MCP-1, MIP-1α and MIG. LIX and MIP-2 were not detected. (**C**) Analysis of changes in CSF expression levels showed that only VEGF was significantly up-regulated 6 h after poly(I:C) treatment. VEGF expression was also significantly different between the 4 p.m. and 10 a.m. time-points. G-CSF was below detection limits. The numbers of animals analyzed were as follows: PBS 6 h, n = 10; poly(I:C) 6 h, n = 10; PBS 24 h, n = 10; and poly(I:C) 24 h, n = 9. Values are presented as the mean ± standard error of the mean. Results obtained in pg/mL were normalized to pg/100 μg of total protein. *P <0.05 between PBS and poly(I:C) groups at 6 h and 24 h post-injection. ^+^*P* <0.05 between 6 h and 24 h after PBS injection. Statistical significance was based on Student’s *t*-test comparisons between groups. ND: not detected.

To examine whether IRSF expression levels show circadian variations, we compared the control samples from 6 h with the samples from the 24 h time-point, which were killed at 4 p.m. and 10 a.m. respectively ( 1). Some IRSF, including IL-5, IL-17, MIP-1β, RANTES, KC, GM-CSF and M-CSF, were not detected in brain homogenates from animals sacrificed at 4 p.m. and became detectable in the samples collected at 10 a.m., but their concentration levels were below 10 pg/100 μg of proteins. Statistical comparisons of the IRSF that were detected at both time-points showed significantly higher concentrations of the pro-inflammatory cytokines IL-1β, IL-7 and IL-9 at 10 a.m. compared with 4 p.m. The largest increase was observed in IL-1β, which changed from 43.9 pg/100 μg at 4 p.m. to 94.9 pg/100 μg of protein at 10 a.m. (Table 2 and Figure 1). The chemokines and CSF eotaxin, MIG and VEGF also showed higher concentration values at 10 a.m., while MCP-1 and MIP-1α had lower values at this time-point as compared with the samples collected at 4 p.m. The largest decline in chemokine concentration was observed in MIP-1α, which dropped from 300 to 154 pg/100 μg of protein (Table 2). Therefore, a subset of IRSF expression appears to be regulated by circadian mechanisms.

Activation of maternal innate immune response by poly(I:C) caused a rapid and significant increase in the pro-inflammatory cytokines IL-7 (205 %) and IL-13 (356 %) 6 h after treatment compared with PBS-treated animals (Table 2 and Figure 1). The cytokines IL-1β, IL-9, IL-15, IL-17 and IL-10 were not substantially affected 6 h after poly(I:C) injection. The concentration levels of the pro-inflammatory cytokines IL-7 and IL-13 remained stable 24 h after injection. By contrast, IL-1β was significantly up-regulated (34 %) at this later time-point, suggesting that the induction of this cytokine required a longer time to develop and is presumably more persistent. It should be noted that IL-1β was the only cytokine significantly up-regulated at 24 h post-poly(I:C) treatment and showed the highest level of expression in fetal brain homogenates (127 pg/100 μg of protein). IL-9 was also present at a high concentration in brain samples (128 pg/100 μg of protein) but its expression levels were unaffected by poly(I:C). Poly(I:C) also induced a significant change in chemokine and CSF concentrations in the fetal brain, including IP-10, MIG, VEGF, MCP-1 and MIP-1α. Interestingly, poly(I:C) induced the up-regulation of the subset of IRSF detected in control fetal brain, indicating that poly(I:C) and/or maternal innate immune activation modulate expression levels of IRSF already present in the fetal brain.

### Poly(I:C) up-regulates different immune response-associated secreted factors in maternal serum and fetal brain

**Figure 2:**
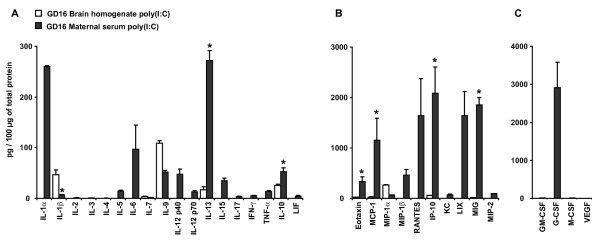
**Comparison of immune response-associated soluble factors concentrations between brain homogenates and maternal serum in poly(I:C) treated animals 6 h after injection.** Values were normalized to total protein content of each tissue. (**A**) Pro- and anti-inflammatory cytokines. IL-1β concentration was significantly lower in maternal serum compared with fetal brain homogenates, while IL-13 and IL-10 were significantly higher. IL-1α, IL-2, IL-3, IL-4, IL-5, IL-6, IL-12(p40), IL-12(p70), IL-15, IL-17, IFN-γ, TNF-α and LIF were not detected in fetal brain. (**B**) Chemokines. Maternal serum showed significantly higher concentration levels of eotaxin, MCP-1, IP-10 and MIG. MIP-1β, RANTES, KC, LIX and MIP-2 were not detected in fetal brain. (**C**) CSF. Only VEGF was detected in both tissues but no significant differences in their concentration levels were observed. *P <0.05 based on Student’s *t*-test comparisons between tissues. Values are expressed as the mean ± standard error of the mean; fetal brain, n = 10; maternal serum, n = 3. ND, not detected.

To examine whether the expression levels of IRSF in the fetal brain followed changes in IRSF concentration in maternal blood, we compared the concentration values of IRSF normalized to total protein concentration in the brain samples with their concentrations in maternal serum 6 h after poly(I:C) treatment (Figure 2). This analysis showed that cytokines that were highly abundant in maternal serum, including IL-1α, IL-6, IL-12(p40) and IL-10 (47 to 309 pg/100 μg of protein), were detected at lower levels (IL-10, 27 pg/100 μg of protein) or not detected (IL-1α, IL-6 and IL-12(p40)) in fetal brain homogenates. By contrast, cytokines IL-1β and IL-9 were detected at high levels in fetal brain samples (47 and 109 pg/100 μg of protein, respectively) while their concentration values were substantially lower in maternal serum (7 and 52 pg/100 μg of protein, respectively). The ten chemokines assayed were also detected at high concentrations in the maternal serum 6 h after poly(I:C) injection (ranging from 65 to 2100 pg/100 μg of protein), while their concentration values in fetal brain homogenates were below 25 pg/100 μg of protein (eotaxin, MCP-1 and MIG) or not detected (MIP-1β, RANTES, KC, LIX and MIP-2). MIP-1α was the only chemokine found at higher levels in fetal brain homogenates (260 pg/100 μg of protein) compared with maternal serum (66 pg/100 μg of protein) and G-CSF concentration in maternal serum reached 2,908 pg/100 μg of protein while it was below detection limits in fetal brain homogenates.

These results indicate that a subset of pro- and anti-inflammatory cytokines and chemokines are normally present in the developing brain and that their expression levels can be modulated directly by poly(I:C) or by the activation of a maternal innate immune response. However, IRSF expression levels in the prenatal brains do not reflect the changes in concentration levels observed in maternal serum, indicating that IRSF expression is modulated by mechanisms within the embryo or the placenta, and are not a passive consequence of the increase in concentration levels in the maternal serum.

### Pre and postnatal brains respond differently to poly(I:C)

To determine whether changes in IRSF expression levels observed in the fetal brain after poly(I:C) injection were unique to the pregnancy environment or could also be triggered after birth, newborn mice were treated with PBS or poly(I:C) on PND4, and IRSF concentrations were measured in brain homogenates 24 h after injection (PND5)(Table 3). As observed in prenatal brain homogenates, a variety of IRSF were detected in control samples, including cytokines IL-1β, IL-2, IL3, IL-6, IL-7, IL-9, IL-13, IL-15, IL-17 and IL-10. The pro-inflammatory cytokines IL-1β and IL-9 showed the highest levels of expression (65 and 130 pg/100 μg of protein, respectively) while the other cytokines were detected at concentrations lower than 22 pg/100 μg of protein. Nine out of the ten chemokines assayed were detected in PND5 control brain samples. Eotaxin, MIP-1α, MIG and IP-10 concentrations ranged from 22 to 81 pg/100 μg of protein, while MCP-1, MIP-1β, RANTES, KC and MIP-2 showed concentration values below 7 pg/100 μg of protein. The four CSF assayed were detectable in PND5 brain homogenates at concentrations below 5 pg/100 μg of protein.

**Table 3:** Cytokine, chemokine and colony stimulating factor concentrations in postnatal brain homogenates

	**24 h after injection**			
	**Control**	**poly(I:C)**	**% change**	**↑ or ↓**
**Pro- and anti-inflammatory cytokines**				
IL-1α	ND	ND		
IL-1β	64.5 ± 4.5	68.1 ± 3.5	6	↑
IL-2	0.8 ± 0.1	1.1 ± 0.1	42	↑*
IL-3	1.1 ± 0.1	2.1 ± 0.3	82	↑**
IL-4	ND	ND		
IL-5	ND	ND		
IL-6	9.5 ± 0.5	7.9 ± 0.8	17	↓
IL-7	6.9 ± 0.5	5.1 ± 0.7	25	↓*
IL-9	129.6 ± 1.8	118.8 ± 4.2	8	↓*
IL-12(p40)	ND	ND		
IL-12(p70)	ND	ND		
IL-13	19.7 ± 1.3	33.1 ± 2.5	68	↑**
IL-15	19.5 ± 0.9	19.3 ± 1.9	1	↓
IL-17	4.6 ± 0.3	5.9 ± 0.5	28	↑*
IFN-γ	ND	ND		
TNF-α	ND	ND		
IL-10	22.3 ± 0.7	21.3 ± 1.7	4	↓
LIF	ND	ND		
**Chemokines**				
Eotaxin	27.8 ± 1.2	33.8 ± 2.7	22	↑*
MCP-1	6.5 ± 0.5	69.4 ± 23.0	962	↑**
MIP-1α	81.4 ± 10.2	83.9 ± 7.2	3	↑
MIP-1β	5.8 ± 0.5	8.5 ± 0.8	47	↑*
RANTES	1.5 ± 0.2	5.1 ± 1.2	235	↑**
IP-10	22.1 ± 1.3	251.0 ± 39.9	1037	↑**
KC	3.3 ± 0.2	4.6 ± 0.6	41	↑*
LIX	ND	ND		
MIG	38.3 ± 1.5	115.3 ± 19.3	201	↑**
MIP-2	4.1 ± 0.3	5.2 ± 0.5	26	↑*
**Colony stimulating factors**				
GM-CSF	4.9 ± 0.4	6.6 ± 0.9	36	↑*
G-CSF	1.4 ± 0.1	1.7 ± 0.2	24	↑*
M-CSF	4.7 ± 0.1	5.2 ± 0.4	11	↑
VEGF	1.1 ± 0.1	0.9 ± 0.1	24	↓*

**Figure 3:**
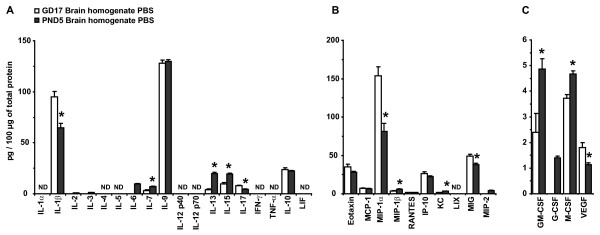
**Comparison of immune response-associated soluble factors expression levels between GD17 and PND5 brain homogenates in phosphate-buffered saline-treated animals show a similar expression profile.** (**A**) Pro- and anti-inflammatory cytokine expression levels were very similar in pre- and postnatal animals. IL-7, IL-13 and IL-15 were significantly higher in PND5 brain homogenates while IL-1β and IL-17 showed lower concentration levels compared with prenatal brains. IL-1α, IL-2, IL-3, IL-4, IL-5, IL-6, IL-12(p40), IL-12(p70), IL-15, IL-17, IFN-γ, TNF-α and LIF were not detected in GD17 brain. (**B**) Chemokines MIP-1α and MIG had lower concentration levels in postnatal compared with prenatal brains, while MIP-1β and KC showed higher concentration levels. LIX and MIP-2 were not detected in GD17 brain homogenates. (**C**) CSF, GM-CSF and M-CSF were significantly higher in postnatal brains while VEGF was significantly lower. G-CSF was not detected in GD17 brain homogenates. Values are expressed as the mean ± standard error of the mean; GD17, n = 10; PND5, n = 11. ND: not detected.

To compare the profile of IRSF expression in postnatal brain homogenates with the one observed in prenatal animals, we plotted the IRSF concentrations normalized to total protein of pre- and postnatal control animals (Figure 3). The profile of IRSF expression observed was remarkably similar between the two age groups. Although variations in the concentrations of cytokines between the two groups were observed, all cytokines detected in prenatal brain homogenates were also present on PND5. A few factors, including IL-2, IL-3, IL-6, MIP-2 and G-CSF were detectable on PND5 and were below detection levels on GD17; however, their concentration levels in PND5 brain homogenates were < 2 pg/100 μg of protein. The concentration levels of IL-7, IL-13, IL-15, MIP-1β, KC, GM-CSF and M-CSF were significantly higher on PND5, while IL-1β, IL-17, MIP1α, MIG and VEGF were significantly lower on PND5 compared with GD17. These results showed that, although there are variations in the concentration levels of IRSF before and after birth, the profile of IRSF expression in the two age groups is similar.

We next examined the response of the postnatal brain to poly(I:C) by administering an i.p. injection of 20 mg/kg of poly(I:C) to each pup on PND4 and determining IRSF concentrations in brain homogenates 24 h after injection (PND5). From the 18 pro- and anti-inflammatory cytokines analyzed, IL-2, IL-3, IL-13 and IL-17 were significantly up-regulated by poly(I:C); however, the concentration levels of IL-2, IL-3 and IL-17 were < 6 pg/100 μg of protein, while IL-13 was 33 pg/100 μg of protein. Eight out of the ten chemokines examined (eotaxin, MCP-1, MIP-1β, RANTES, IP-10, KC, MIG and MIP-2) were significantly up-regulated by poly(I:C) treatment. MCP-1 and IP-10 showed the highest levels of increase, of 962 % and 1,037 % respectively. The CSF GM-CSF and G-CSF were also significantly up-regulated in the postnatal brain by poly(I:C) but their expression values were below 7 pg/100 μg of protein (Table 3).

**Figure 4:**
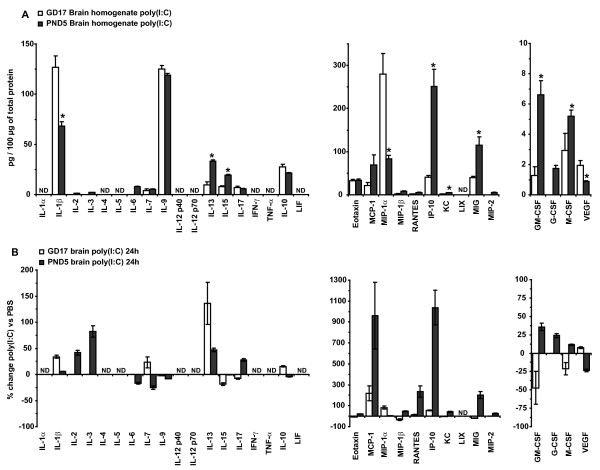
**Analysis of the effect of poly(I:C) treatment on immune response-associated soluble factors expression levels between gestational day 17 and postnatal day 5 brain homogenates.** (**A**) Comparison of IRSF concentrations normalized to total protein content. A significantly lower concentration of the cytokine IL-1β was detected in postnatal brain while IL-13 and IL-15 were significantly higher. The chemokine MIP-1α and the CSF VEGF were reduced in postnatal brain while IP-10, KC, MIG, GM-CSF and M-CSF were significantly higher. (**B**) Comparison of the percentage change in IRSF concentration levels induced by poly(I:C) between pre- and postnatal brain homogenates 24 h after treatment. Note the higher percentage changes in chemokines and CSF in the postnatal brain compared with less pronounced differences in cytokines. Values are mean ± standard error of the mean. *P <0.05 based on Student’s *t*-test comparisons. GD17, n = 9; PND5, n = 14. ND, not detected.

To examine whether the profile of IRSF in brain homogenates induced by innate immune activation was similar between pre and postnatal animals, we compared the IRSF concentrations and the percentage change between GD17 and PND5 brain homogenates 24 h after poly(I:C) injection (Figure 4). While IL-1β was significantly up-regulated by poly(I:C) at GD17, the concentration levels remained almost unchanged at PND5. In contrast, IL-2, IL-3 and IL-17 were significantly up-regulated in response to poly(I:C) treatment in PND5 brain homogenates and were unchanged in GD17 animals. IL-13 was the only cytokine up-regulated at both developmental stages although the percentage change was larger in the fetal brain. More pronounced differences in the response between the two groups were observed in chemokine and CSF concentration levels. While only MCP-1 and MIP-1α were up-regulated in prenatal brains, eotaxin, MCP-1, MIP-1β, RANTES, IP-10, KC, MIG, MIP-2, GM-CSF and G-CSF were significantly up-regulated in PND5 brain samples. These analyses indicate that, although the expression profile of IRSF in pre- and postnatal brains under physiological conditions was quite similar, the effect of innate immune activation by poly(I:C) on IRSF concentrations in brain homogenates was substantially different between the two ages. Indeed, the profile of IRSF response in PND5 brains was more similar to the changes observed in maternal serum after poly(I:C) injection than to the ones observed in fetal brain homogenates. This evidence supports the view that the environment of the embryo generates a unique response to poly(I:C), either directly or secondary to maternal innate immune activation, triggering the up-regulation of a subset of IRSF, including IL-1β, that may affect CNS development and cause long-lasting behavioral abnormalities.

## Discussion

The aim of this study was to examine the profile of IRSF expression in the fetal brain after maternal innate immune activation using the synthetic analogue of viral dsRNA poly(I:C) to induce the innate immune response, and to determine whether innate immune activation in early postnatal life affects IRSF levels in the pup’s brain. The goal of this analysis was to identify IRSF produced by the innate immune response to viral infections that could undermine normal brain development and impair the acquisition of cognition and social behaviors later in life.

We choose to mimic a viral infection instead of the most frequently used bacterial mimic agent lipopolysaccharide (LPS), because viral infections during pregnancy are common during the influenza season and appear to predispose the offspring to develop psychiatric illness [10,13]. Intravenous and i.p. administration of poly(I:C) are widely used as inducers of the innate immune response, which mimics the first phase of defensive mechanisms against viral infections [30,31]. The structure of poly(I:C) resembles the structure of dsRNA generated in host cells during viral replication, and it is recognized by Toll-like receptors that activate the innate immune response [23,38]. The use of poly(I:C) as an innate immune response activator is advantageous because it avoids the use of infectious agents within the working environment, and treatments can be standardized, facilitating comparisons between experiments and between laboratories. However, it should be noted that i.v. or i.p. injection of poly(I:C) do not exactly reproduce a natural viral infection, as viruses most commonly infect epithelial cells of the respiratory and digestive tracts, and very rarely infect and replicate in the blood stream or in the peritoneum. Therefore, the immune cells activated by i.v. or i.p. administration of poly(I:C) are likely to be different than the ones activated during an ordinary viral infection. Activation of immune cells in different tissues may lead to the generation of an innate immune response with different profiles of cytokine production. Thus, the profile IRSF in maternal serum observed in the present study after i.p. administration of poly(I:C) may not be exactly the same as the profile observed after an i.v. injection of poly(I:C) or after a common viral infection. Nevertheless, the profile of IRSF detected in fetal brain homogenates did not correlate with the profile observed in maternal serum, indicating that IRSF production was regulated within the fetus or in the placenta. This suggests that, regardless of the route of administration, the effect of poly(I:C) on IRSF expression levels in fetal brains is expected to be similar to the effect caused by a natural viral infection.

Maternal exposure to poly(I:C) close to the end of the mouse pregnancy (GD16 to GD17) impairs associative and reversal learning, exploration in open field and social behavior of the offspring. In addition, an increase in anxiety and a decrease in pre-pulse inhibition have been observed in post-pubertal animals exposed to poly(I:C) during pregnancy, indicating that innate immune activation by poly(I:C) during the final period of the pregnancy affects CNS development and causes long-lasting impairments of animal behaviors associated with psychiatric symptoms [28,40-42]. The developmental stage of the mouse CNS at GD16 correlates with GD68 to GD94 of human brain development, depending on whether cortical (GD93.3), limbic (GD68.4) or non-cortical/limbic events (GD73.7) are compared [34-36]. At this developmental stage, neurogenesis is completed in most brain structures, and it coincides with the time of axonal innervation and synapse formation in the cerebral cortex [34]. Perturbation of CNS development at GD16 is unlikely to cause severe morphological malformations, but rather abnormalities associated with the formation and establishment of neuronal circuits. Thus, the impairment in social behavior and the appearance of psychiatric symptoms observed in animals exposed to poly(I:C) on GD16 to GD17 may be caused by the increase in pro-inflammatory cytokines observed in the present study, which may impinge on axonal growth and synapse formation resulting in defective CNS wiring and/or establishment of neuronal connections.

PND4 in mouse brain development correlates with GD107 to GD147 in humans, also depending on the brain structure compared (cortical GD146.7, limbic GD106.9, or non-cortical/limbic GD115.4) [34,35]. At this developmental stage, neuronal circuits are still forming and therefore are vulnerable to epigenetic insults. The effect of poly(I:C) injection in PND4 pups showed that activation of the innate immune response in early postnatal life predominantly induces the expression of chemokines and CSF instead of the pro-inflammatory cytokines observed in prenatal animals. However, the impact of postnatal immune activation on cognition and social behavior after puberty has not been examined. This analysis will facilitate the identification of periods of higher vulnerability to infections by establishing whether the IRSF induced by maternal innate immune activation or by the innate immune response in postnatal pups have either protective or noxious effects on brain development and animal behavior in the adult.

From the 18 pro- and anti-inflammatory cytokines examined in this study, IL-1β, IL-7 and IL-13 were significantly up-regulated in prenatal brains, while IL-2, IL-3 and IL-13 were found up-regulated in the postnatal brain. However, the low concentrations values of IL-2, IL-3 and IL-7 suggest that the changes observed in the concentration levels of these three cytokines may not affect CNS development, unless the increase occurs in particular brain structures. IL-1β had the highest expression levels in prenatal brain and showed a significant increase 24 h after poly(I:C) injection. Although an increase in IL-1β concentration was also detected in maternal serum, the concentration levels of IL-1β normalized to total protein were approximately 16 times higher in the fetal brain as compared to maternal serum. High levels of IL-1β were also detected in the brains of PND5 animals; however, the concentration values were not affected by poly(I:C) treatment. These results suggest that the increase of IL-1β in the fetal brain caused by poly(I:C) derives from the fetal brain itself and/or placental tissues as it has been observed in animals exposed to maternal innate immune activation by LPS [43,44]. A recent study examined the effect of experimental genital mycoplasmosis in pregnant rodents on cytokines expression levels by injecting *Mycoplasma pulmonis* into GD14 pregnant rats and analyzing cytokine expression levels on GD18 [45]. The study found significantly higher levels of the cytokine IL-1β in amniotic fluids and a significant increase in IL-1β mRNA expression levels in the pup’s brains. These results are in agreement with the findings reported in the present study and further highlight the possible role of IL-1β in the long-lasting behavioral deficits observed in animals exposed to maternal innate immune activation during gestation.

In adult rodents, both IL-1β and its receptor IL-1R1 are expressed in various brain regions, including the cerebral cortex, hippocampus and hypothalamus [46-51], and IL-1β expression levels are affected by innate immune activation [52-58]. Similarly, maternal exposure to LPS increased the level of IL-1β mRNA in the fetal rat and mouse brain; however, contradictory results have been reported at the protein level (reviewed in [32]). Pregnant mice injected with poly(I:C) showed different effects in IL-1β expression levels depending on the day of gestation and sampling time after the injection [28]. In agreement with this previous report [28], we found no changes in IL-1β concentration in the fetal brain 6 h after poly(I:C) injection on GD16. However, the increase in IL-1β gene expression levels observed after poly(I:C) administration on GD17 [28] may account for the increase in IL-1β concentration at the protein levels that we observed 24 h after poly(I:C) treatment on GD16. These results indicate that, although IL-1β expression levels may vary, a mimic of viral infections significantly increases IL-1β expression levels in the fetal brain for at least 24 h after the activation of an innate immune response.

IL-1β mediates a variety of host acute responses to infection, including fever, loss of appetite and somnolence [59-61]. Expression of both IL-1β and its receptor IL-1R1 in the CNS is regulated in various pathological conditions, including brain injury, inflammation and neurodegeneration [62], suggesting that IL-1β contributes to pathological mechanisms observed in these disorders. However, physiological levels of IL-1β expression in the CNS of young animals appears to be necessary for learning and memory consolidation, while the increase in IL-1β observed in normal aging appears to impair these neurological functions [51,63]. Indeed, administration of IL-1β to young adult rodents impairs learning and memory acquisition [64-66], fear conditioning [67], exploratory behavior [68,69], mating [70], sleep [71] and appetite [72], indicating that IL-1β has a number of cellular targets that regulate CNS function. Binding of IL-1β to the IL-1R1 induces formation of a signaling complex with the IL-1 receptor accessory protein, which are both members of the Toll-like receptor family of proteins [73,74]. In the CNS, activation of IL-1R1 by IL-1β activates signaling pathways that modulate intracellular calcium [16], expression of neurotransmitter receptors [16], activation of cAMP response element-binding (CREB) [75] and brain-derived neurotrophic factor (BDNF) expression [76]. Thus, abnormal activation of these signaling pathways during neural development by IL-1β can impinge on a variety of developmental mechanisms, resulting in the perturbation of cell migration, axonal growth and synapse formation and undermining cognitive, emotional and behavioral performance later in life. These developmental defects may enhance the sensitivity of the CNS to the effects of genetic abnormalities and traumatic (physical or emotional) factors, and increase the risk for developing mental illness.

The concentration levels of IL-1β in the fetal brain 24 h after PBS injection were approximately two times higher than the ones observed 6 h after the injection. Although IL-1β concentration levels in fetal brain may vary during development, the most likely explanation for this difference is a circadian variation. Animals at the 6 h time-point were killed 4 p.m. (close to the end of the light period), while the ones corresponding to the 24 h time-points were killed at 10 a.m. Similar variations of IL-1β concentration in adult rat brain tissue have been observed at the protein and mRNA level [50,77], and these variations have been attributed to circadian changes in glucocorticoids concentration. Glucocorticoids suppress IL-1β transcription and mRNA stability [78], and the increase in pro-inflammatory cytokine mRNA levels in the brain after immune activation is enhanced in adrenalectomized animals [50,79]. These results suggest that the variation in IL-1β concentration that we observed in control subjects between two different times of the day was likely due to physiological circadian variations of IL-1β that may be regulated by glucocorticoids.

The increase in IL-13 expression levels in both prenatal and postnatal brain homogenates is interesting because IL-13 is primarily produced by activated mast cells. Murine mast cells express TLR-3 and are strongly activated upon treatment with poly(I:C) [80]. Mast cells are of hematopoietic origin but they are able to enter the brain under normal and pathological conditions [81]. In our study, the expression levels of IL-13 in maternal serum were significantly increased 6 h after poly(I:C) treatment, suggesting that mast cells may have been activated very early after treatment in the periphery. In addition, the increased levels of MCP-1 detected in both fetal and postnatal brains may enhance the recruitment of mast cells [82]. Mast cells form close interactions with neurons and transfer intracellular content by transgranulation, which may modulate neuronal functions [83] and affect CNS development. Whether higher expression levels of IL-13 correlates with a massive colonization of activated mast cells in the fetal brain remains to be determined; however, autistic patients often present ‘allergy-like’ symptoms in the absence of elevated serum IgE, suggesting that non-allergic mast cell activation commonly occurs in these patients [84].

The effect of maternal poly(I:C) injection on the expression levels of pro- and anti-inflammatory cytokine IL-6, IL-10 and TNF-α in the fetal brain has been previously examined [85]. In the present study, IL-6 was undetectable in fetal brains in both control- and poly(I:C)-treated samples. However, IL-6 was detected in maternal serum and in PND5 brain homogenates, and its concentration in maternal serum was increased after poly(I:C) administration on GD16, indicating that IL-6 participates in maternal innate immune activation and may mediate deleterious effects in embryo development as has been previously reported [21]. In addition, we found that TNF-α and IL-10 concentrations in the fetal brain were unaffected by poly(I:C) treatments, indicating that our results are overall consistent with previous studies [28].

A significant up-regulation by poly(I:C) of chemokines MCP-1, MIP-1α, IP-10 and MIG was detected in the fetal brain, while a larger repertoire of chemokines was up-regulated in the postnatal brain after poly(I:C) injection on PND4 (eotaxin, MCP-1, MIP-1β, RANTES, IP-10, KC, MIG and MIP-2). Chemokines contribute to normal brain development by providing cues for the migration of newly generated neurons and glial cells and modulate axon path-finding [20]. In addition, MCP-1 (also known as chemokine CC motif ligand 2) enhances neuronal excitability and synaptic transmission in hippocampal neurons [86]. MCP-1 and its cognate receptor chemokine CC motif receptor 2 are constitutively expressed in various brain regions [87] and modulate neuronal physiology [88,89], suggesting that its up-regulation in the fetal brain can affect the formation of neuronal circuits. Finally, VEGF contributes to various mechanisms of CNS development including neuronal migration, differentiation and axonal growth and path-finding [90-94], indicating that deregulation of VEGF expression during critical periods of brain development may perturb neuronal migration and formation of neural circuits. Thus, the increase in chemokine and CSF expression in the developing brain by the innate immune response provides additional mechanisms that may play a role in pathogenic events associated with maternal immune activation, with long-lasting impact on brain function.

## Conclusions

This study provided a comprehensive analysis of IRSF expression in fetal and postnatal brains after innate immune activation by the synthetic analogue of viral dsRNA poly(I:C). This analysis revealed that a subset of pro-inflammatory cytokines (IL-1β and IL-13), chemokines (MCP-1, MIP-1α, IP-10 and MIG) and the CSF VEGF are significantly up-regulated in the fetal brain during maternal innate immune activation. In addition to their role in the innate immune response, these factors participate in neural development and neuronal physiology, indicating that their abnormal up-regulation as a consequence of maternal innate immune activation may affect CNS formation and increase the risk for developing psychiatric illness later in life. Further studies are required to examine the regulation of IRSF by maternal innate immune activation in specific brain structures affected in psychiatric disorders with presumed neurodevelopmental origin, including the cerebral cortex and caudate/putamen/accumbens nuclei, and to determine the role of particular IRSF in abnormal behaviors observed in psychiatric illnesses.

## Abbreviations

Bp, Base pair; CSF, Colony stimulating factor; CNS, Central nervous system; dsRNA, Double-stranded RNA; G-CSF, Granulocyte colony stimulating factor; GD, Gestational day; GM-CSF, Granulocyte-macrophage colony stimulating factor; IFN, Interferon; IgE, Immunoglobulin E; IL, Interleukin; i.p., Intraperitoneal; IP-10, Interferon inducible protein 10; IRSF, Immune response-associated secreted factor; KC, Keratinocyte derived chemokine; LIF, Leukemia inhibitory factor; LIX, Lipopolysaccharide induced CXC chemokine; LPS, Lipopolysaccharide; MCP-1, Monocyte chemoattractant protein 1; M-CSF, Macrophage colony stimulating factor; MIG, Monokine induced by gamma-interferon; MinDC, Minimum detection concentration; MIP, Macrophage inflammatory protein; PBS, Phosphate-buffered saline; PCR, Polymerase chain reaction; poly(I:C), Polyriboinosinic-polyribocytidylic acid; PND, Postnatal day; RANTES, Regulated on activation normal T cells expressed and secreted; TLR3, Toll-like receptor 3; TNF, Tumor necrosis factor; VEGF, Vascular endothelial growth factor.

## Competing interests

The authors declare that they have no competing interests.

## Authors’ contributions

GAB participated in the experimental design, animal treatments, IRSF measurements using multiplexed bead-based immunoassay (Milliplex Map) and processing in a Luminex 100 IS instrument, sex determination, data analysis, and preparation of the manuscript. JLB conceived the study and participated in the design and coordination of the study, brain dissection and sample processing, data processing and analysis, and organizing and drafting the manuscript. GAB and JLB have read and approved the final submitted version of this manuscript.
